# How Heme Oxygenase-1 Prevents Heme-Induced Cell Death

**DOI:** 10.1371/journal.pone.0134144

**Published:** 2015-08-13

**Authors:** Lilibeth Lanceta, Jacob M. Mattingly, Chi Li, John W. Eaton

**Affiliations:** Molecular Targets Program, James Graham Brown Cancer Center, University of Louisville, Louisville, Kentucky, United States of America; North Carolina State University, UNITED STATES

## Abstract

Earlier observations indicate that free heme is selectively toxic to cells lacking heme oxygenase-1 (HO-1) but how this enzyme prevents heme toxicity remains unexplained. Here, using A549 (human lung cancer) and immortalized human bronchial epithelial cells incubated with exogenous heme, we find knock-down of HO-1 using siRNA does promote the accumulation of cell-associated heme and heme-induced cell death. However, it appears that the toxic effects of heme are exerted by “loose” (probably intralysosomal) iron because cytotoxic effects of heme are lessened by pre-incubation of HO-1 deficient cells with desferrioxamine (which localizes preferentially in the lysosomal compartment). Desferrioxamine also decreases lysosomal rupture promoted by intracellularly generated hydrogen peroxide. Supporting the importance of endogenous oxidant production, both chemical and siRNA inhibition of catalase activity predisposes HO-1 deficient cells to heme-mediated killing. Importantly, it appears that HO-1 deficiency somehow blocks the induction of ferritin; control cells exposed to heme show ~10-fold increases in ferritin heavy chain expression whereas in heme-exposed HO-1 deficient cells ferritin expression is unchanged. Finally, overexpression of ferritin H chain in HO-1 deficient cells completely prevents heme-induced cytotoxicity. Although two other products of HO-1 activity–CO and bilirubin–have been invoked to explain HO-1-mediated cytoprotection, we conclude that, at least in this experimental system, HO-1 activity triggers the induction of ferritin and the latter is actually responsible for the cytoprotective effects of HO-1 activity.

## Introduction

The impetus for the present investigations was a paper published over a decade ago by Yachie et al. [1] on the first human with heme oxygenase-1 (HO-1) deficiency. In their description of this unique patient, the authors reported a phenomenon that was difficult to explain. When they challenged an Epstein-Barr virus-transformed lymphoblastoid cell line from this patient with exogenous heme (50–200 μM), over 24 hours most or nearly all of these cells died. In contrast, a similarly immortalized line from a donor with normal HO-1 activity was completely unaffected. This, despite the fact that in neither case was there a significant change in the amounts of heme in the culture medium over the incubation period.

These observations raised the question of the nature of the heme toxicity. This question was partially answered by Fortes and colleagues [2] who concluded that, in murine macrophages, heme caused necrotic cell death and the latter required both Toll like receptor 4 (TLR4)-dependent production of tumor necrosis factor (TNF) and reactive oxygen species (ROS) generated in a TLR4-independent manner. The involvement of heme-mediated necrosis was further supported by the observation that addition of necrostatin-1 (an inhibitor of receptor-interacting protein 1; RIP1) largely prevented heme-induced cell death.

Here, we add to these earlier results using A549 (human lung adenocarcinoma) and immortalized human bronchial epithelial (HBEC) cells as a model. We find, as expected, that siRNA knock-down of HO-1 sensitizes both cell types to heme cytotoxicity. In A549 cells, heme-mediated cytotoxicity is accompanied by increased intracellular ROS generation and lysosomal rupture. Heme-induced lysosomal rupture is prevented by pre-incubation with desferrioxamine, implicating free intralysosomal iron in the process of cell death. Furthermore, the involvement of ROS (specifically, H_2_O_2_) in heme-mediated cell death is supported by experiments in which 3-amino-1,2,4-triazole (3-AT) was used to inhibit catalase resulting in increased cell death. siRNA knock-down of catalase had a similar effect.

However, our results do not agree with those of Fortes et al. [2] in as much as knock-down of TLR4, rather than affecting signaling and TNF production, decreases heme uptake into target cells. This indicates that TLR4 may also function as a conduit facilitating heme uptake. The decreased heme uptake decreases heme-mediated ROS generation but in A549 cells–which are not professional phagocytes–has no effect on the induction of TNF-alpha mRNA. Finally, in this cell model, cell death is not affected by addition of necrostatin-1 indicating the RIP-1 is not involved.

## Materials and Methods

### Reagents

Unless otherwise specified, reagents were purchased from Sigma-Aldrich (St. Louis, MO). Ferritin heavy chain (FTH1) antibody RabMAb, HO-1 antibody and catalase antibody were purchased from Abcam (Cambridge, MA). Toll like receptor 4 antibody was from Santa Cruz Biotechnology (Santa Cruz, CA). Protease Inhibitor Cocktail Set V, EDTA-free was from Calbiochem (Darmstadt, Germany). HO-1 siRNA (ID#194530), catalase siRNA (ID#2444) and toll like receptor 4 siRNA (ID#14195) were purchased from Applied Biosystems (Carlsbad, CA). Lipofectamine RNAimax was purchased from Invitrogen (Grand Island, NY). Necrostatin-1 was purchased from Selleckchem (Houston, TX). Alamar blue was from AbD Serotec (Raleigh, NC). 2’,7’–Dichlorofluorescein diacetate (DCFDA) was purchased from Molecular Probes (Carlsbad, CA).

### Cell culture

We employed A549 cells (human lung adenocarcinoma), which was acquired from American Type Culture Collection. The cells were grown in T75 flasks in Ham's-F12 medium (Gibco) supplemented with 10% fetal bovine serum in a humidified incubator with 5% CO_2_ at 37°C. The cells were split every other day by resuspension in fresh medium and inoculated at 1 x 10^6^ into new T-75 flasks. Immortalized human bronchial epithelial cells (HBEC) were cultured in keratinocyte serum-free medium (KSF) supplemented with 5 ng/mL epidermal growth factor and 50 μg/mL bovine pituitary extract. Cells were grown at 37°C in a humidified atmosphere with 5% CO2.

### HO-1 knockdown with siRNA

A549 cells were cultured on 12 well plates (4 x 10^4^/well) and grown overnight under standard culture conditions. Transfection with 10 nM HO-1 siRNA was done in optiMEM for 24 hours. RNAimax was used as a transfection reagent. Changes in HO-1 expression were determined by western blot and enzymatic assay. HO-1 activity was measured on isolated microsomes from A549 wild type cells and HO-1 knockdown cells using a protocol described by Zhang, et al. [3] with slight modification. HBEC cells were cultured in a 12 well plates (7 x 10^4^/well) and grown overnight under standard culture conditions. Transfection with 5 nM HO-1 siRNA was done in KSF medium. After 4 hours the medium was replaced with fresh KSF medium. After 24 hours the cells were challenged with 25 μM heme. After another 24 hours cell viability was measured using Alamar blue reduction. Changes in HO-1 expression were determined by western blot.

### Lysosomal integrity

A549 cells were plated in a 12 well plate, 4 x 10^4^ cells/well. After 24 hours, HO-1 siRNA transfection was performed (or not) and the cells were incubated for a further 24 hours. After 24 hours of heme exposure (prior to detectable cell death), lysosomal integrity was estimated by staining with 5 μg/mL acridine orange (AO) for 15 minutes. The cells were then detached with trypsin and lysosomal integrity was estimated by flow cytometry [4].

### Involvement of intracellular iron and endogenous H_2_O_2_ in heme-induced cell death

To test the importance of intralysosomal iron in heme-mediated killing of HO-1 deficient cells, A549 cells were plated in 12 well plates, 4 x 10^4^/well and cultured for 24 hours. Following this, the cells were transfected with 10 nM HO-1 siRNA for 24 hours. The cells were then treated with 1 mM DFO for 2 hours after which the medium was replaced and the cells were challenged with 100 μM heme. Cell viability was measured 36 hours later by alamar blue reduction and lysosomal integrity was measured by AO staining of the lysosomal compartment as described above.

To assess the possible involvement of endogenously generated H_2_O_2_ in heme-driven cytotoxicity, A549 cells were plated in 12 well plates (4 x 10^4^/well) for 24 hours and then transfected (or not) with 10 nM HO-1 siRNA for a further 24 hours. Cells were then pre-treated with 5 mM 3-amino-1,2,4-triazole (which inhibits catalase activity) for 2 hours. Following these treatments, the medium was changed and the cells were exposed to 100 μM heme for 36 hours. Cell viability was measured by alamar blue reduction.

### Knockdown of Toll Like Receptor 4 (TLR4)

An earlier report [2] suggested that TLR4 might be important in mediating heme toxicity. To investigate this, A549 cells were cultured on 12 well plates (4 x 10^4^/well) and grown overnight under standard culture conditions. Transfection with 10 nM TLR4 siRNA ± 10 nM HO-1 siRNA was done for 24 hours. Changes in TLR4 expression were determined by western blot, and following 36 hours exposure to 100 μM heme, cell viability was assessed using alamar blue.

### ROS measurement

A549 cells were plated in 12 well plates, 4 x 10^4^cells/well. Cells were grown overnight under normal conditions. The cells were transfected (or not) with both 10 nM HO-1 and TLR4 siRNA for 24 hours. After 24 hours of transfection, the medium was removed and changed to fresh media. Then the cells were challenged with or without 100 μM heme. After 24 hours, cells were detached with 0.05% trypsin. Cells were washed with 1X PBS (+calcium) twice. The cells were re-suspended in 10 μM DCFDA and incubated at 37°C for 30 minutes. Oxidized DCFDA was estimated using a Becton Dikenson FACS calibur.

### Heme uptake

A549 cells were plated in 10 cm plates (7 x 10^5^/plate) and grown overnight under normal culture conditions. Transfection with 10 nm HO-1 and TLR4 siRNA was done for 24 hours. Cells were challenged with 100 μM heme for 6 hours (a time at which no cell death had occurred). Cells were lifted with trypsin and washed twice with phosphate buffered saline. Samples were lysed using snap freezing and thawing. Cell-associated heme was assayed as previously described [5].

### Necrostatin-1

An earlier report [6] indicated that necrostatin-1 (an inhibitor of RIP1) blocked heme-induced cell death in murine macrophages. We tested this in control and HO-1 knockdown A549 cells plated in 12 well plates (4 x 10^4^/well) and grown overnight under normal conditions. Transfection with 10 nM HO-1 siRNA was carried out as above and after 24 hours 50 μM necrostatin-1 was added for 10 minutes and then the cells were challenged with 100 μM heme for 36 hours. Cell viability was assessed with alamar blue.

### TNF-alpha

A549 cells were plated in 24 well plates (2 x 10^4^/well) and grown overnight under normal conditions. Transfection with 10 nM HO-1 and TLR4 siRNA was performed for 24 hours. Then the cells were challenged with 100 μM heme for 24 hours (prior to detectable cell death). RNA isolation was performed using RNeasy (Qiagen; Valencia, CA) and qPCR was performed to measure TNF-alpha mRNA expression using a probe from Applied Biosystems.

### Ferrostatin-1

Because the mode of cell death bore some resemblance to the previously reported “ferroptosis” [6], we investigated whether this was involved in heme-mediated cell death. A549 cells were grown in 12 well plates (4 x 10^4^/well) and grown 24 hours under normal conditions. Transfection of 10 nM HO-1 siRNA was done for a further 24 hours. The cells were then pre-incubated with 0, 0.25, 0.5, and 1.0 μM of ferrostatin-1 for 30 minutes and challenged with 100 μM heme for 36 hours and cell viability was assessed using alamar blue.

### Measurement of bound and 'loose' iron

For these experiments, A549 cells were plated in 10 cm plates (7 x 10^5^/plate). Twenty-four hours later, HO-1 siRNA transfection was done for 24 hours. The cells were exposed to 100 μM heme for another 24 hours. The cells were washed once with Chelex pre-treated PBS, removed from the plates by gentle scraping and transferred to 15 ml tubes. Samples were centrifuged at 4,000 x g for 5 minutes, supernatants were aspirated and 500 μl cold 10% perchloric acid was added. Following transfer of the samples into 1.5 ml tubes for 30 minutes incubation on ice, the samples were centrifuged for 5 minutes at 12,000 x g (4°C) and the supernatant was assayed for 'loose' iron. Three hundred μl of 50% nitric acid was added to the pellets and incubated at 60°C overnight for measurements of ‘bound’ iron. These samples were neutralized with 10 N NaOH. Both fractions were assayed for iron using ferene S as previously described [7]. We should note that no perfect test for ‘bound’ and ‘loose’ iron exists but this particular technique was developed using red cells. Despite the iron-rich nature of these cells we found very low background levels of ‘loose’ iron [7]. The ‘bound’ iron reported here is likely in heme, iron sulfur clusters and ferritin.

### Co-transfection of HO-1 siRNA and FTH1 plasmid DNA

A549 cells were cultured on 6 well plates (seeded at 3 x 10^5^ cells/well) and grown 24 hours under standard culture conditions. Transfection with HO-1 siRNA and FTH1 plasmid DNA was done in optiMEM for 24 hours, using Lipofectamine 2000 for transfection reagent. The cells were challenged with 100 μM heme for 36 hours and cell viability was measured by alamar blue. Changes in HO-1 and FTH1 expression were determined by western blot. The cells were transfected with HO-1 siRNA and FTH1 plasmid DNA and incubated for 24 hours. The cells were then challenged with 100 μM heme for 24 hours following which the cells were harvested and protein was isolated for western blot.

## Results

**Fig 1:**
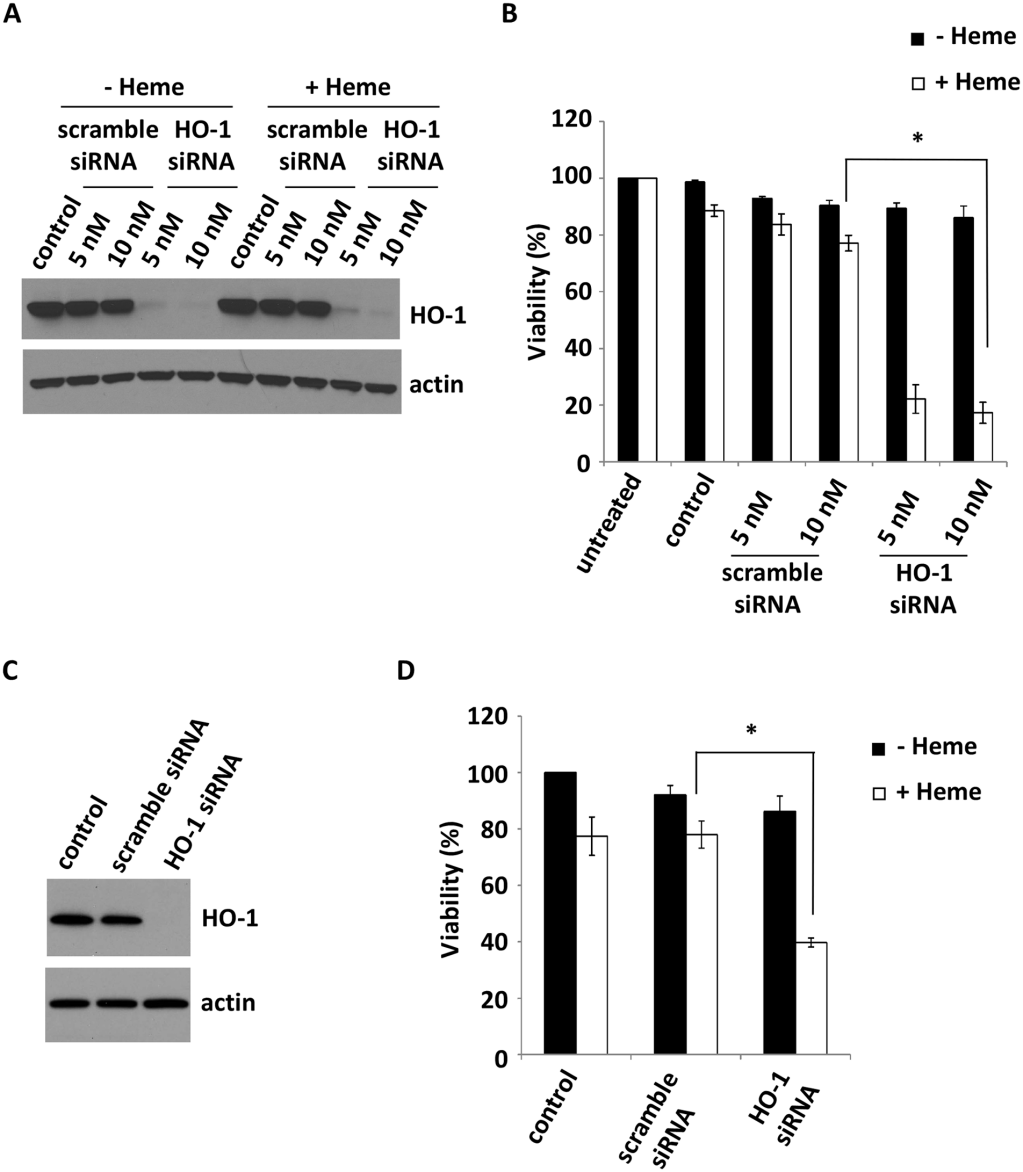
HO-1KO in A549 cells sensitizes the cells to heme-mediated cytotoxicity. **(A)** A549 cells were transfected with the indicated HO-1 siRNA for 24 hours, then treated with or without 100 μM heme for 36 hours. Western blot shows substantial reduction in HO-1 mRNA expression in cells transfected with HO-1 siRNA. **(B)** Cells were exposed to 100 μM heme for 36 hours and viability was assessed with Alamar blue reduction. Data are means ± SEM of 6 independent experiments. *p≤0.01. **(C)** Human bronchial epithelial cells (HBEC) were transfected with the indicated HO-1 siRNA for 24 hours, and HO-1 expression was measured by western blot. **(D)** HBEC were transfected with the indicated HO-1 siRNA for 24 hours, then treated with or without 25 μM heme for 24 hours. Cell viability was measured by Alamar blue reduction.

In agreement with other reports (including that of Yachie [1] mentioned above), as shown in Fig 1A and 1B decreased HO-1 (hereafter, HO-1KO) activity greatly increases heme-mediated cell death. Direct measurements of HO-1 activity in the siRNA treated cells indicated that activity was reduced by ~95% compared to untreated cells or cells exposed to scrambled siRNA (results not shown). To ensure that the effects of HO-1KO were not restricted to A549 cells, we conducted similar experiments with immortalized human bronchial epithelial cells. These cells were immortalized by the introduction of two genes, *hTERT* and *Cdk4* [8]. Once again, HO-1KO increased the cytotoxic effects of heme exposure (Fig 1C and 1D) although in this case lesser concentrations of heme were required to induce significant cell death. This is likely due to higher steady state expression of HO-1 in A549 cells (also typical of many types of cancers).

**Fig 2:**
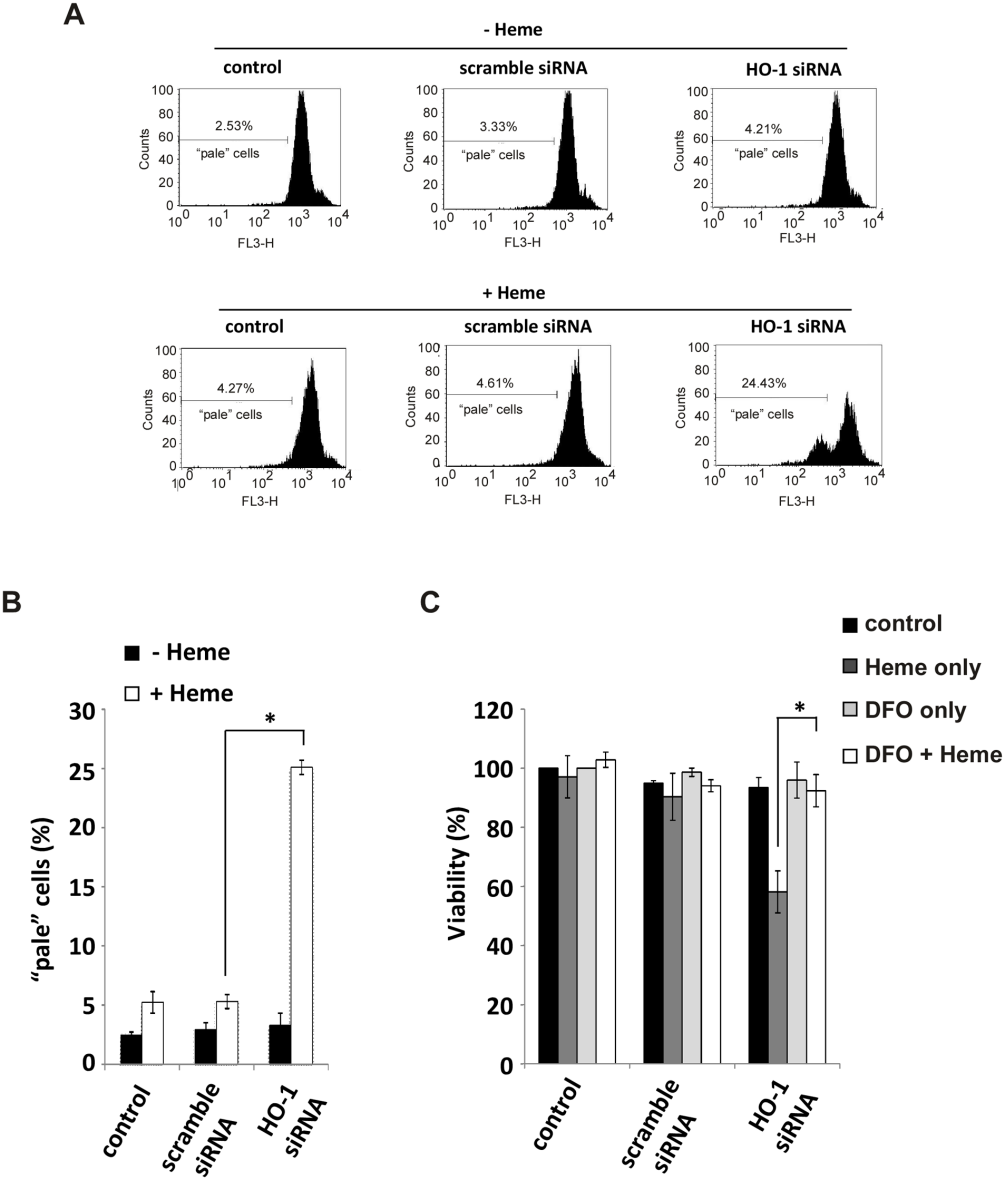
Heme exposure of HO-1KO A549 cells leads to lysosomal rupture and loss of viability which is prevented by pre-incubation with DFO. A549 cells were challenged with 100 μM heme for 24 hours and then stained with 5 μg/ml AO. **(A)** A549 cells deficient in intact lysosomes (‘pale’ cells) were measured by flow cytometry. **(B)** The summary of data shown in (A). Data are means ± SEM of 3 independent experiments. *p≤0.01. **(C)** A549 cells were pre-treated with 1 mM DFO for 2 hours and then challenged with 100 μM heme for 24 hours. Cell viability was measured using alamar blue. Data are means ± SEM of 3 independent experiments. *p≤0.05.

In A549 cells with HO-1KO, cell death appears to involve lysosomal rupture. Following 24 hours exposure to heme, cells were incubated briefly with acridine orange (AO). AO, a metachromatic fluorophore and a lysosomotropic base, concentrates by proton trapping (as AOH+) primarily inside lysosomes which then fluoresce as orange dots when excited with blue light [4]. In Fig 2A and 2B, ‘pale’ cells refers to cells which have lost the ability to concentrate AO due to lysosomal instability or rupture [4]. Clearly, HO-1KO sensitizes cells to lysosomal damage following heme exposure.

The nature of cell death bears some resemblance to the previously described ‘ferroptosis’, an iron-dependent form of nonapoptotic cell death [6]. As in this earlier report, we find that the lysosomal rupture and death of HO-1KO cells is prevented by pre-incubation of cells with DFO (Fig 2C). This suggests that lysosomal destabilization arises from intralysosomal iron because DFO does not readily cross the plasma membrane but rather enters into the lysosomal compartment via fluid phase endocytosis [4,9,10].

**Fig 3:**
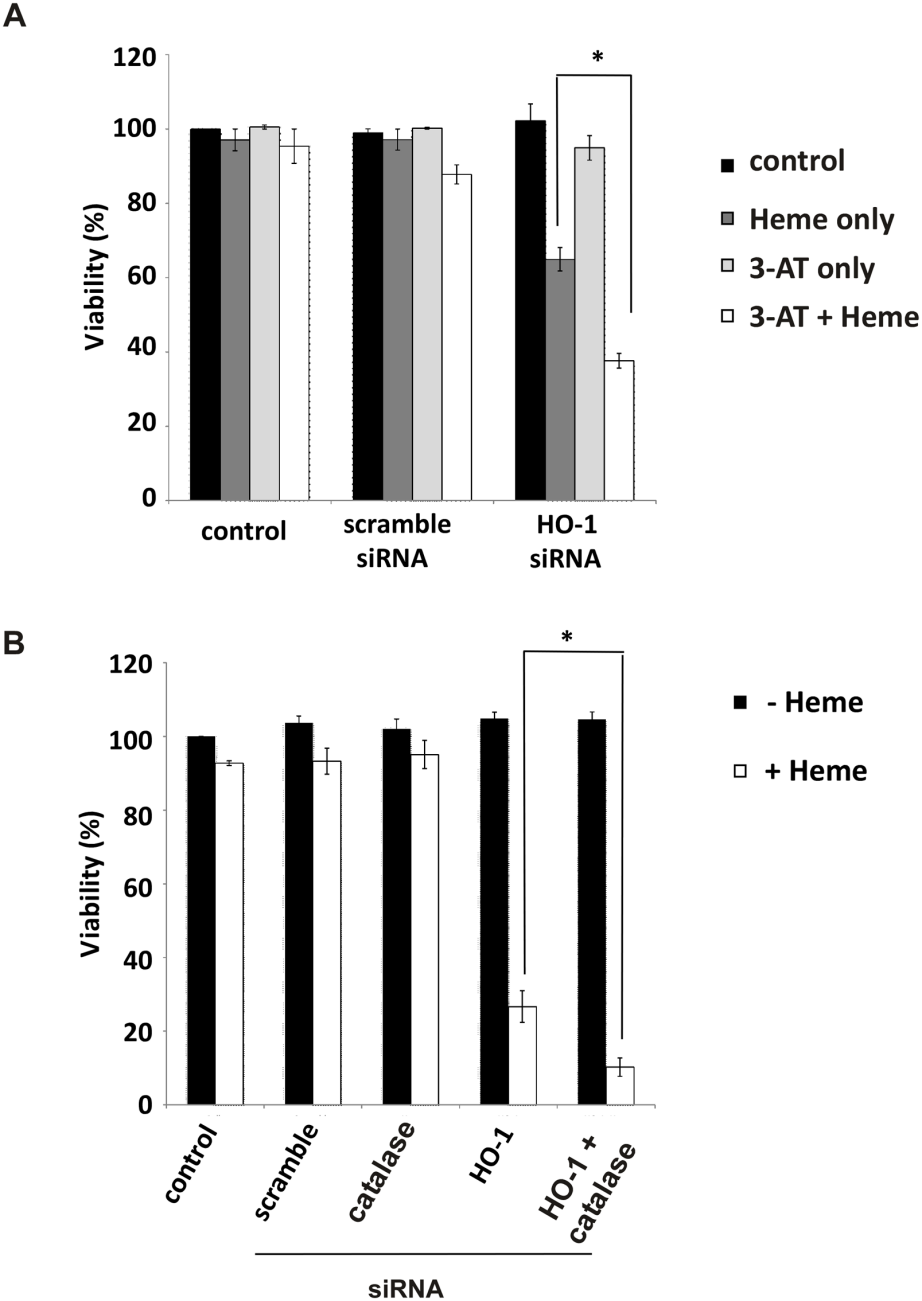
Chemical and siRNA-mediated inhibition of catalase activity in A549 cells enhances the cytotoxic effects of heme. **(A)** A549 cells were pre-treated with 5 mM 3-AT for 2 hours and then challenged with 100 μM heme for 36 hours. Cell viability was measured by alamar blue. **(B)** A549 cells were transfected with both 10 nM HO-1 and human catalase siRNAs for 24 hours and then challenged with 100 μM heme for 36 hours. Cell viability was measured by alamar blue. In both cases, data are means ± SEM of 3 independent experiments. *p≤0.05.

The rupture of iron-loaded lysosomes requires both redox active intralysosomal iron and either exogenous or cell-generated oxidants [4,9,10]. The involvement of cell-generated intracellular H_2_O_2_ is supported by experiments in which cells were pre-incubated with 3-AT which inhibits catalase activity and, therefore, H_2_O_2_ clearance. While HO-1KO cells were sensitive to killing by heme, cell death was significantly enhanced in cells pre-incubated with AT (Fig 3A). Because AT could have off-target actions, we also used siRNA to decrease catalase activity which similarly sensitized HO-1KO cells to heme (Fig 3B).

**Fig 4:**
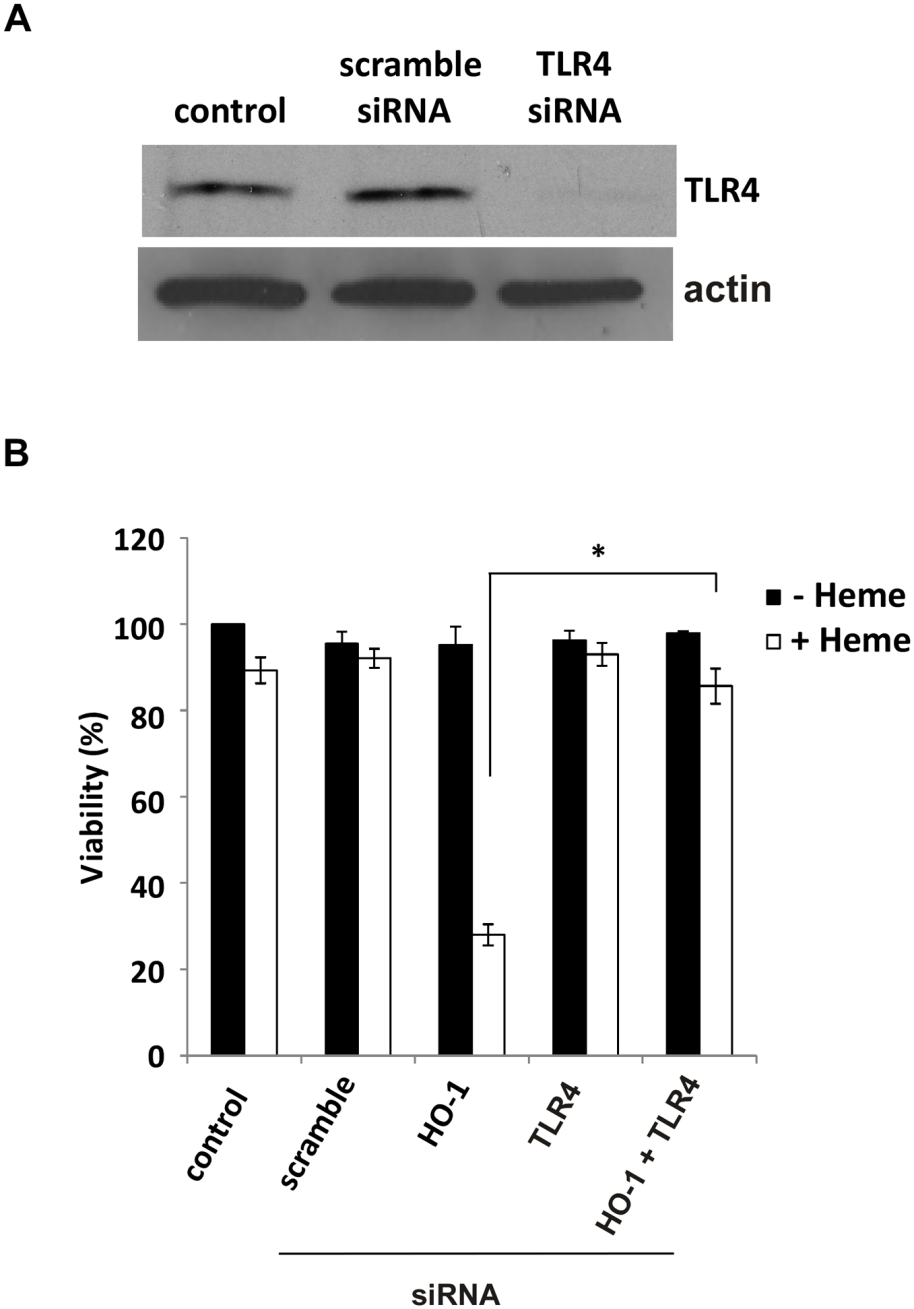
siRNA-mediated knockdown of TLR4 suppresses heme-induced cytotoxicity in A549 cells. **(A)** Western blot showing successful knockdown of TLR4 after 24 hour exposure to 10 nM TLR4 siRNA. **(B)** TLR4 knockdown prevents the cytotoxic effects of 36 hour exposure of A549 cells to 100 μM heme (viability assessed by alamar blue). Data are means ± SEM of 3 independent experiments.*p≤0.01.

**Fig 5:**
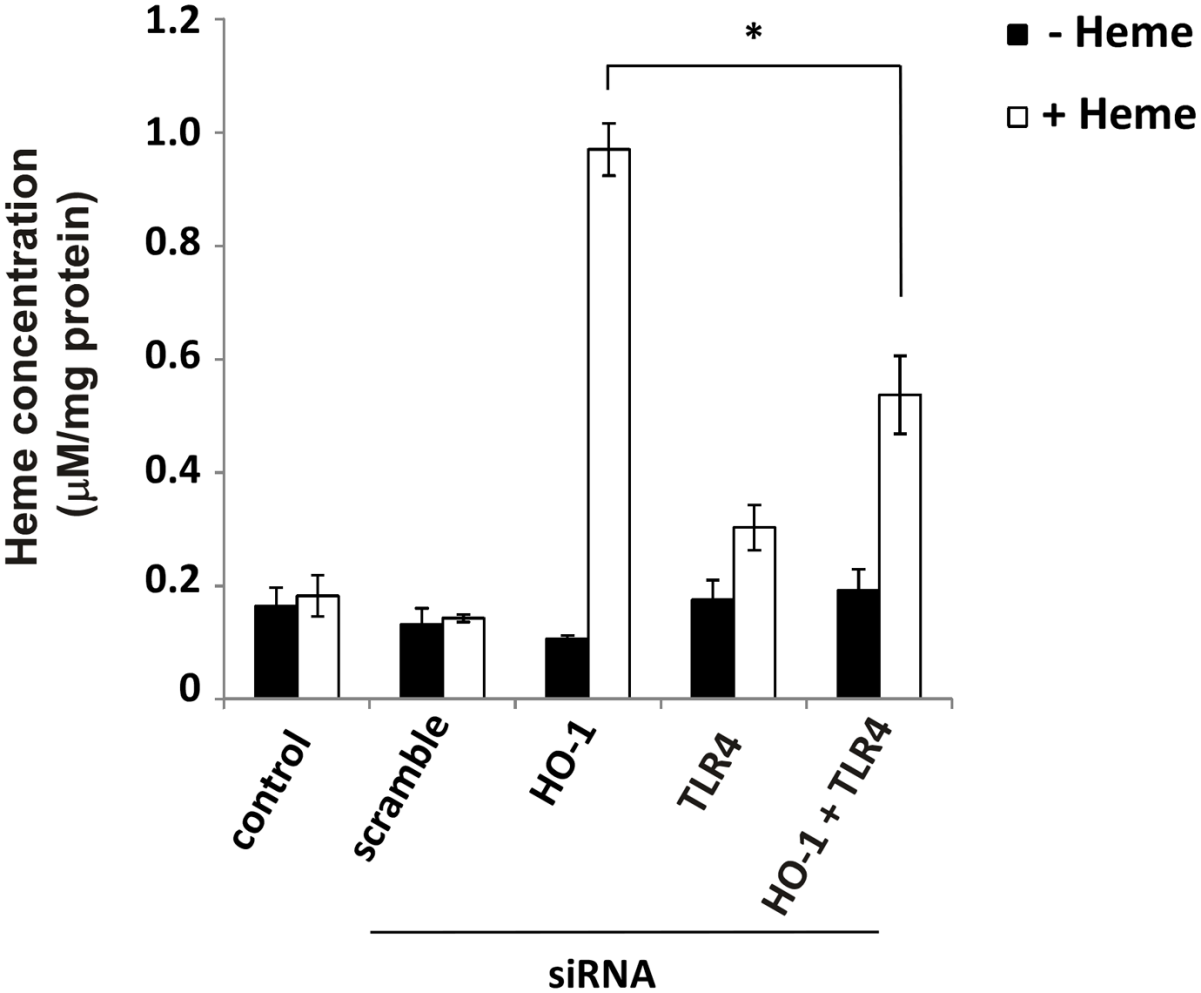
TLR4 knockdown suppresses heme accumulation in HO-1KO A549 cells. Following 24 hour transfections with both 10 nM TLR4 and HO-1 siRNAs, the cells were challenged with 100 μM heme for 6 hours, detached with 0.05% trypsin, washed twice and lysed using snap freezing and thawing. Heme was measured spectrophotometrically. Data are means ± SEM of 3 independent experiments. *p≤0.01.

We confirmed the previously reported [2] involvement of TLR4 in heme-mediated killing of HO-1KO cells. As shown in Fig 4, cell death is abrogated in HO-1KO cells in which TLR4 has also been knocked down. However, in contrast to the results reported by Fortes et al. [2], this may have more to do with decreased heme import as opposed to intracellular signaling events. HO-1KO cells accumulate large amounts of heme during control incubations but knock down of TLR4 in these cells decreases heme accumulation by >50% (Fig 5).

**Fig 6:**
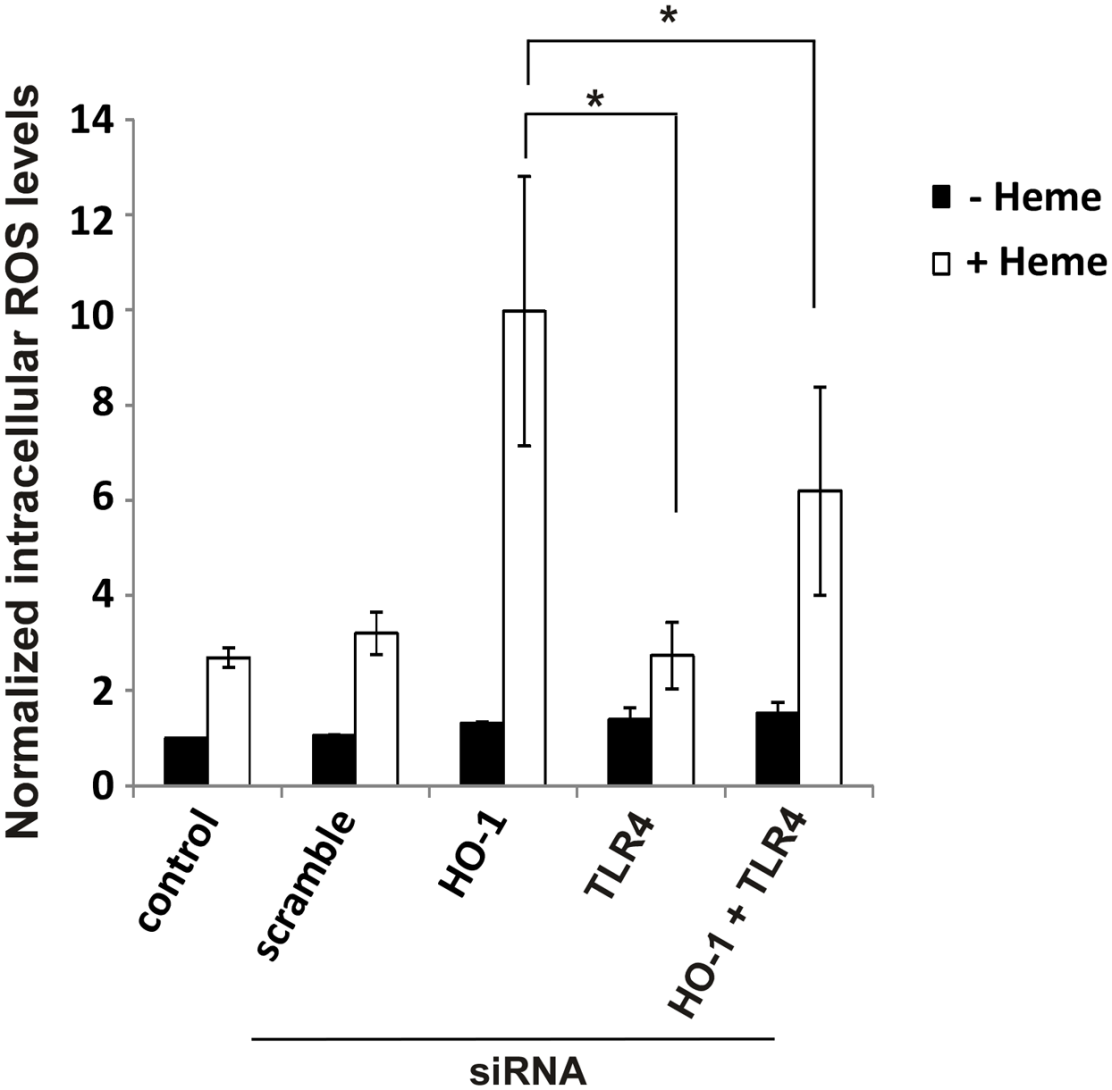
Knockdown of TLR4 in A549 cells suppresses heme-induced intracellular ROS generation. Following 24 hour transfection with both HO-1 and TLR4 siRNAs the cells were challenged with 100 μM heme for a further 24 hours. The cells were then detached with 0.05% trypsin and stained with 10 μM DCFDA at 37°C for 30 minutes. ROS was measured using flow cytometry. Data are means ± SEM of 3 independent experiments. *p≤0.05.

**Fig 7:**
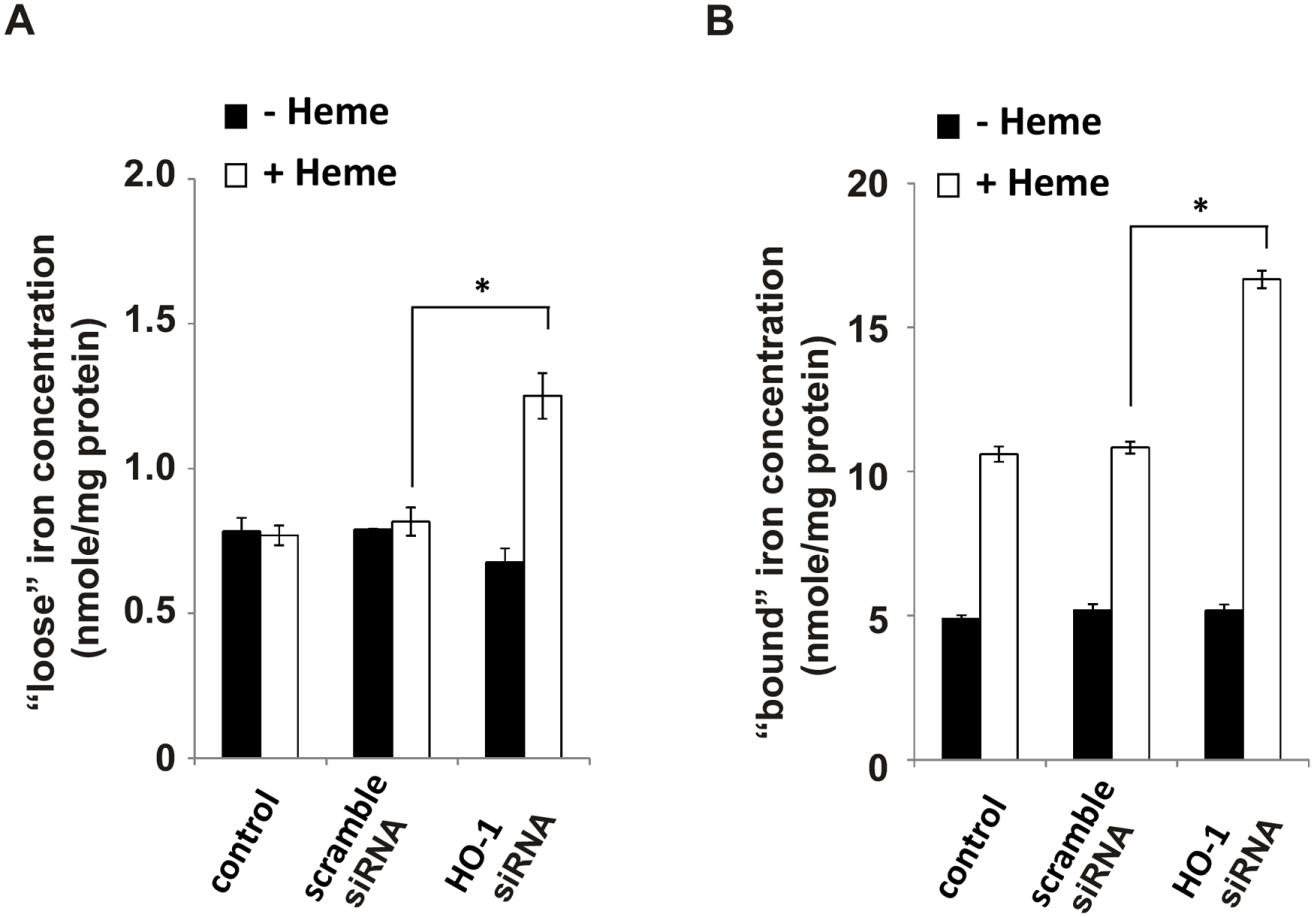
HO-1KO A549 cells accumulate increased amounts of “loose” and total intracellular iron. The cells were transfected with HO-1 siRNA for 24 hours and then challenged with 100 μM heme for a further 24 hours. The cells were harvested by scraping and extracted with cold 10% perchloric acid. **(A)** Concentration of “loose” iron in the extract supernatants. **(B)** “Bound” intracellular iron concentrations. Note that in a nitric acid digest, “bound” iron includes not just ferritin bound iron but also heme iron. Data are means ± SEM of 3 independent experiments. *p≤0.01.

The lesser heme uptake in cells treated with TLR4 siRNA is accompanied by significant decreases in intracellular ROS generation as measured by 2’,7’–dichlorofluorescein diacetate (DCFDA) oxidation (Fig 6). Although most investigators assume increased DCF oxidation is a generalized indicator of ‘ROS’ production, Karlsson and colleagues have shown that this oxidation requires peroxidase activity (either redox active iron or cytochrome c released into the cytoplasm) [11]. Indeed, measurements of total intracellular iron following 24 hour incubation of cells with 50 μM heme reveals large increases of ‘loose’ iron in HO-1KO cells following 24 hour incubation with heme (Fig 7). It should be noted that in HO-1KO cells, this ‘loose’ iron likely derives from oxidative scission of the heme ring rather than HO-1 activity [12].

Contrary to the earlier report by Fortes et al. [2], we find no evidence of transcriptional up-regulation of TNFalpha mRNA following heme exposure of either control or HO-1KO A549 cells whereas cells exposed to LPS alone showed substantial up-regulation of TNF-alpha mRNA (results not shown). Furthermore, heme-mediated cell death is unaffected by the addition of necrostatin-1 (a specific inhibitor of RIP1) (results not shown). This indicates that, in the case of A549 cells, RIP1-mediated necrosis is not involved.

The heme-induced death of HO-1KO cells bore some similarity to the previously reported “ferroptosis” [6]. This form of cell death requires that the target cells express RAS, is mediated by agents such as erastin and is prevented by ferrostatin-1 and analogues thereof. However, although A549 cells do have activated RAS, we find that the addition of ferrostatin has no effect on heme-induced death of HO-1KO A549 cells (results not shown).

**Fig 8:**
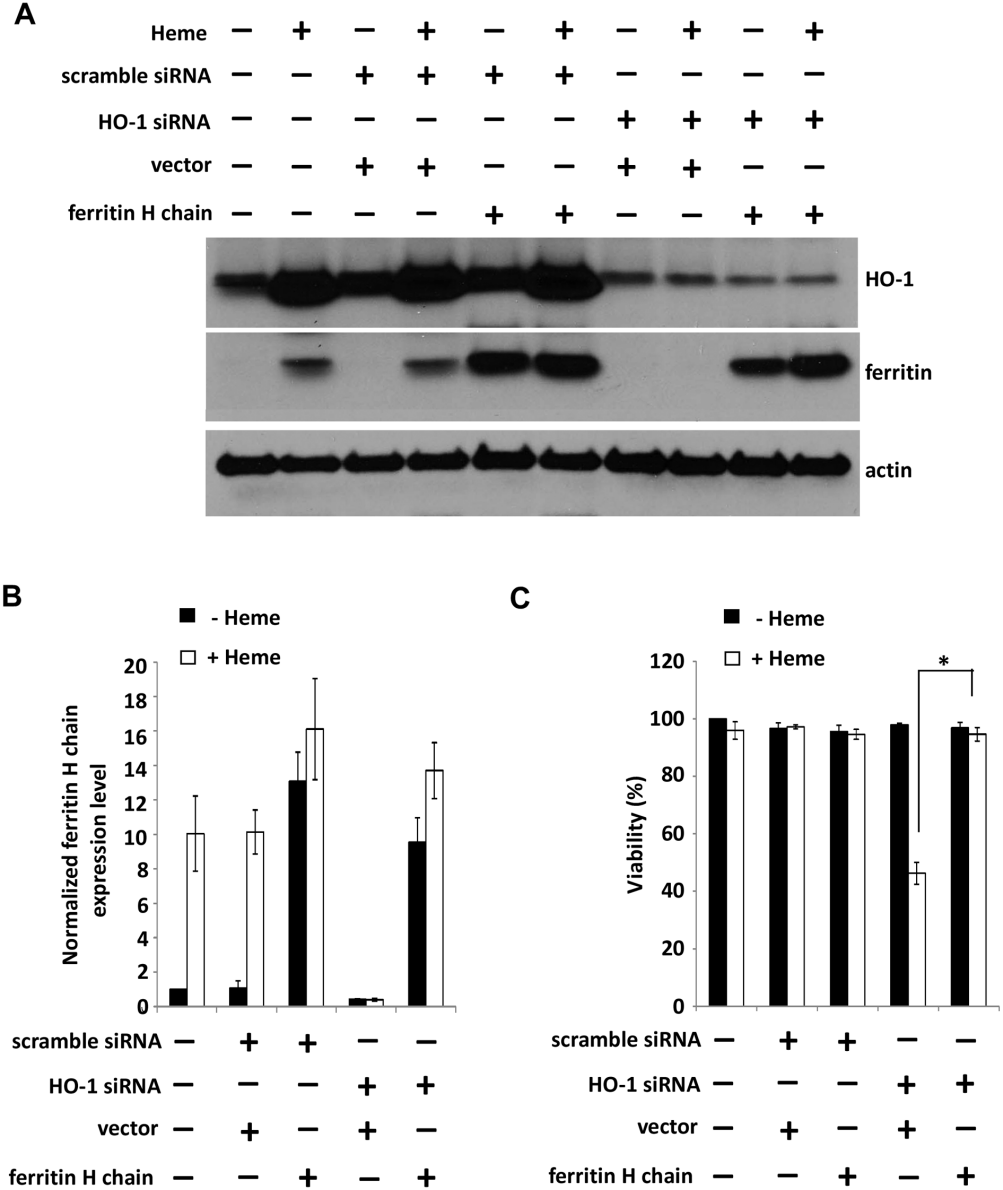
HO-1KO blocks heme-mediated induction of ferritin and compensatory over-expression of ferritin H chain prevents heme-mediated cytotoxicity. **(A)** A549 cells were transfected with 10 nM HO-1 siRNA and western blot indicates pronounced ferritin H chain induction in control cells and those transfected with scrambled siRNA but not HO-1 siRNA. **(B)** The summary of normalized ferritin H chain levels shown in (A). Quantification of ferritin H chain expression normalized to actin (n = 3 independent experiments). **(C)** Over-expression of ferritin H chain in HO-1KO A549 cells blocks heme-mediated cytotoxicity. A549 cells were transfected with 2 μg of FTH1 expressing pCMV6-ferritin for 24 hours (± HO-1 siRNA) and then challenged with100 μM heme for 36 hours. Cell viability was measured with alamar blue. Data are means ± SEM of 3 independent experiments. *p≤0.01.

Finally, we return to the original question of why HO-1KO cells should be readily killed by heme while wild-type cells are highly resistant. In partial answer, we find that wild-type cells exposed to heme upregulate ferritin ~10 fold. However, similarly treated HO-1KO cells show almost no ferritin induction (Fig 8A). It appears that the lack of ferritin induction is not a consequence of HO-1 knockdown per se because HO-1KO cells exposed to ferric ammonium citrate (200 μM) for 24 hours show similar ferritin induction compared to wild-type cells (2.2 x increase vs. 2.1 x increase respectively normalized to beta actin, n = 3).

Given that heme exposed HO-1KO cells have higher levels of ‘loose’ iron, the lack of ferritin induction is a surprising observation and suggests a presently unknown linkage between HO-1 activity, ‘loose’ iron sensing and the induction of ferritin. Regardless of the nature of this linkage, we find that over-expression of ferritin H chain in HO-1KO cells provides substantial protection against heme-induced cell death (Fig 8C) as might be anticipated from our earlier observations [13].

## Discussion

The importance of HO-1 in protection against heme toxicity was graphically illustrated by the severity of vascular (and other) disorders in the first human found to have HO-1 deficiency [1]. It is further supported by experiments in which Epstein-Barr virus-transformed lymphoblastoid cells from this patient were exposed to heme. The cells were highly sensitive to heme toxicity while similarly immortalized HO-1 expressing cells were unaffected [1]. Inasmuch as there were no changes in heme concentration over a 24 hour period of incubation with either cell type, these early results raised the question of the nature of heme toxicity and of exactly how HO-1 might block this.

Here, we have affirmed the preferential cytotoxic effects of heme on HO-1KO cells. This was true of both A549 (human lung cancer) cells and immortalized human bronchial epithelial cells. However, we should note that the heme concentrations which caused cell death in A549 cells were substantially higher than those causing the death of the immortalized HBEC cells. This almost certainly reflects the well known upregulation of HO-1 in various types of cancer cells.

In another example of heme toxicity in HO-1^-/-^ cells, Kovtunovych et al. reported that macrophages from HO-1 knock-out mice died following erythrophagocytosis while macrophages from wild type mice were unaffected [14]. It is likely that the death of HO-1 knock-out macrophages was caused by heme released from hemoglobin from the phagocytosed red cells.

Additional observations in this regard were made by Fortes et al. [2] who also tested macrophages from HO-1KO mice exposed to exogenous heme. In these cells, heme caused necrotic cell death. These authors found that cell death required both TLR4-dependent production of TNF as well as reactive oxygen species (ROS) generated in a TLR4-independent manner. The involvement of heme-mediated necrosis was further supported by the observation that addition of necrostatin-1 (a specific inhibitor of RIP1) largely prevented heme-induced cell death.

In the present investigations, we have confirmed the involvement of TLR4 in heme-mediated death of HO-1KO A549 cells. However, perhaps because these lung cancer cells are not professional phagocytes, we did not observe concomitant up-regulation of TNF expression. Rather, it appears that TLR4 facilitates the import of heme and knock-down of TLR4 both suppresses intracellular heme accumulation as well as consequent cell death.

Our results suggest that, in HO-1KO cells, heme itself is not directly toxic. Instead, it appears that once lodged within the cell, heme iron is released and the latter is the important toxic principle. (In this regard, it is important to note that heme degradation does not require HO-1 activity; heme is readily destroyed when exposed to pro-oxidant environments [12]). Moreover, it would seem that the ‘loose’ iron in question may be within lysosomes. The chelator DFO enters cells via fluid phase endocytosis and resides almost exclusively within the lysosomal apparatus [4,9,10]. Pre-incubation of HO-1KO cells with DFO protects against heme toxicity which supports the idea that intralysosomal ‘loose’ iron is the important cytotoxin. Furthermore, the toxicity of this iron is amplified by ROS–especially hydrogen peroxide—generated by the target cells; chemical or siRNA inhibition of cellular catalase activity sensitizes HO-1KO cells to heme-mediated cytotoxicity.

At present, we have no clear explanation for why ferritin is readily induced in HO-1 wild type cells exposed to heme while little or no ferritin induction occurs in HO-1KO cells (despite the accumulation of much more ‘loose’ iron in the latter). This issue was partially addressed by Gozzelino and Soares in a recent review [15]. In their view, ferritin heavy chain expression restrains TNF-dependent JNK activation which latter will predispose to apoptotic cell death. Given the lack of TNF induction in our system, this explanation would not fit the current results. Regardless of the answer to this question, we conclude that in this experimental system the cytoprotective effects of HO-1 are due to the induction of ferritin synthesis rather than to HO-1 activity per se. This is supported by our observations that overexpression of ferritin H chain in HO-1KO cells protects against heme-mediated cell death while knock-down of ferritin H chain in wild type cells leads to increased heme cytotoxicity.

The cytoprotective effects of HO-1 have been variously ascribed to the generation of CO [16] or the ‘antioxidant’ bilirubin [17]. Indeed, there are numerous publications supporting both possibilities. However, as argued elsewhere [13], in many cases this may be unlikely because the amounts of available heme in most cells may be insufficient to generate much of these two products and both will readily diffuse from areas in which they are generated. Overall, we suggest that in many circumstances, the cytoprotective effects of HO-1 may more likely be explained by linked induction of ferritin.
